# Highly Effective Protocol for Differentiation of Induced Pluripotent Stem Cells (iPS) into Melanin-Producing Cells

**DOI:** 10.3390/ijms222312787

**Published:** 2021-11-26

**Authors:** Maciej Sułkowski, Marta Kot, Bogna Badyra, Anna Paluszkiewicz, Przemysław M. Płonka, Michał Sarna, Dominika Michalczyk-Wetula, Fabio A. Zucca, Luigi Zecca, Marcin Majka

**Affiliations:** 1 Department of Transplantation, Faculty of Medicine, Institute of Pediatrics, Jagiellonian University Medical College, 30-663 Cracow, Poland; maciek.sulkowski@uj.edu.pl (M.S.); marta.kot@uj.edu.pl (M.K.); bogna.badyra@gmail.com (B.B.); ania.dubiel@uj.edu.pl (A.P.); 2 Department of Biophysics, Faculty of Biochemistry, Biophysics and Biotechnology, Jagiellonian University, 30-387 Cracow, Poland; przemyslaw.plonka@uj.edu.pl (P.M.P.); michal.sarna@uj.edu.pl (M.S.); dominika.michalczyk@uj.edu.pl (D.M.-W.); 3 Institute of Biomedical Technologies, National Research Council of Italy, 20054 Milan, Italy; fabio.zucca@itb.cnr.it (F.A.Z.); luigi.zecca@itb.cnr.it (L.Z.)

**Keywords:** melanin, induced pluripotent stem cells (iPS), differentiation protocol, UV light, melanization disorders

## Abstract

Melanin is a black/brown pigment present in abundance in human skin. Its main function is photo-protection of underlying tissues from harmful UV light. Natural sources of isolated human melanin are limited; thus, in vitro cultures of human cells may be a promising source of human melanin. Here, we present an innovative in vitro differentiation protocol of induced pluripotent stem cells (iPS) into melanin-producing cells, delivering highly pigmented cells in quantity and quality incomparably higher than any other methods previously described. Pigmented cells constitute over 90% of a terminally differentiated population and exhibit features characteristic for melanocytes, i.e., expression of specific markers such as MITF-M (microphthalmia-associated transcription factor isoform M), TRP-1 (tyrosinase-related protein 1), and TYR (tyrosinase) and accumulation of black pigment in organelles closely resembling melanosomes. Black pigment is unambiguously identified as melanin with features corresponding to those of melanin produced by typical melanocytes. The advantage of our method is that it does not require any sophisticated procedures and can be conducted in standard laboratory conditions. Moreover, our protocol is highly reproducible and optimized to generate high-purity melanin-producing cells from iPS cells; thus, it can serve as an unlimited source of human melanin for modeling human skin diseases. We speculate that FGF-8 might play an important role during differentiation processes toward pigmented cells.

## 1. Introduction

Invented in 2006 by Shinya Yamanaka et al., induced pluripotent stem (iPS) cells [1] opened a new chapter in the potential application of stem cells in regenerative medicine for many disorders. Since iPS cells can be acquired by reprogramming somatic cells of any type, appropriate iPS-derived somatic cells can recapitulate phenotypes of disease in vitro [2,3,4], thus representing not only a personalized drug screening platform but also an exceptional model for studying pathomechanisms of diseases [5].

Melanin is a dark pigment found predominantly in the skin whose main role is photo-protection [6]. It is produced in specialized cells called melanocytes, within which the melanin is contained in dedicated membranous organelles called melanosomes [7].

Knowledge of human melanin structure, function, and synthesis is limited mainly due to scarce sources of the pigment. Among these sources, invasive skin biopsies and hair samples are the most common; however, they are also limited and may deliver melanin in a highly photo-degraded and dehydrated state, as the skin and hair of adult donors have been exposed to a lifetime of sunlight and are deprived of water [6].

A promising approach to acquiring high amounts of melanin is the use of in vitro cell cultures. Unfortunately, culture, expansion, maturation, and, most importantly, pigmentation of melanocytes in vitro are troublesome enough without mentioning the obstacles regarding the acquisition of explants for primary cultures as the first step. A solution might come from efficient in vitro differentiation of stem cells—of iPS cells in particular.

Indeed, there are protocols of piPS (protein induced pluripotent stem cells) differentiation into melanin-producing melanocytes [8,9,10] in which piPS cells through a multistep process are stimulated by many agents to differentiate into cells with melanocyte characteristics. However, none of the described protocols deliver high amounts of pure, non-degraded human melanin, as acquired cells are only fairly pigmented. The protocol described below was developed from a classic method of embryonic stem cell (ESC) differentiation into dopaminergic neurons [11], which share ectoderm differentiation fate and a common progenitor with melanocytes. Quite unexpectedly, the differentiated cells were heavily pigmented; therefore, we decided to investigate the nature of the observed phenomenon.

We present here a novel and robust protocol for in vitro differentiation of piPS cells which delivers human melanin in abundance and quality previously unmet in melanin-producing cell cultures. It should be noted that our protocol is highly efficient and repetitive and does not require sophisticated laboratory procedures (e.g., genetic modifications), techniques, or reagents. Pigmented cells constitute about 90% of a terminally differentiated population in each independent culture. The in vitro-acquired pigment is comparable to the quality and quantity of melanin produced in a natural way by melanocytes.

## 2. Results

### 2.1. The Induced Pluripotent Stem Cell Differentiation into Pigment-Containing Cells

**Figure 1:**
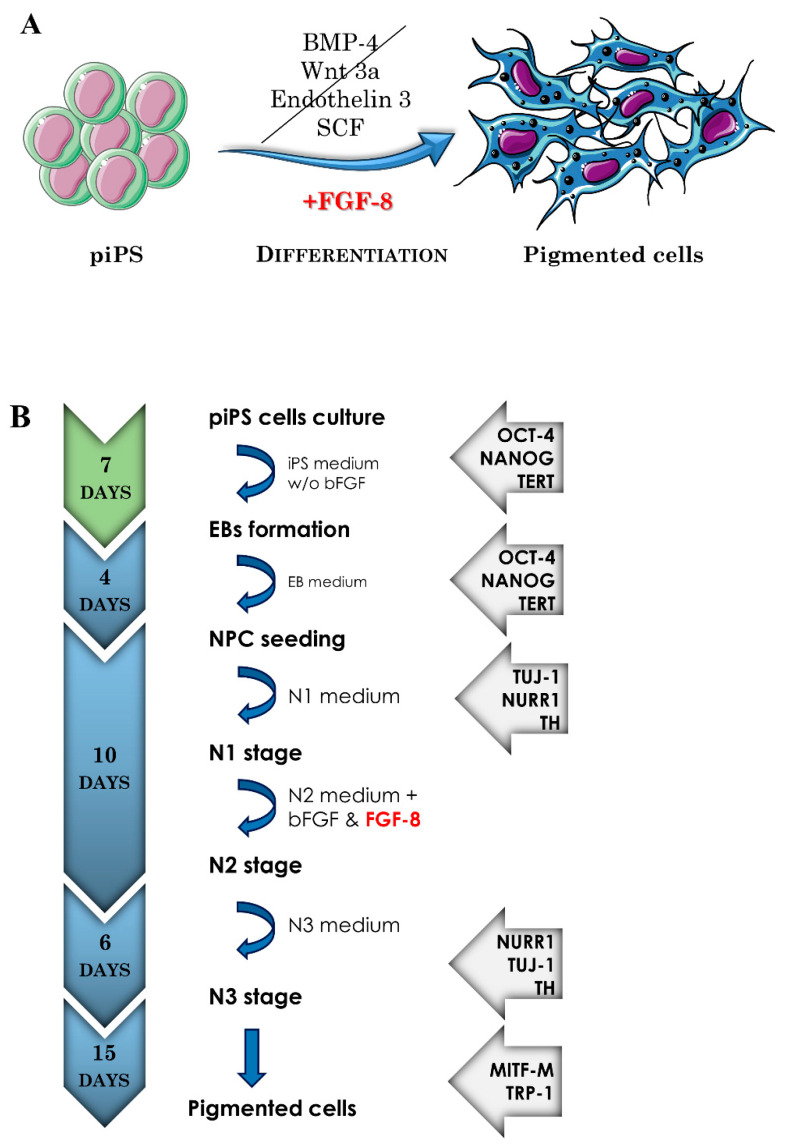
(**A**) Graphical illustration of the differentiation of piPS (protein induced pluripotent stem cells) into melanin-producing cells. The developed protocol involves the use of FGF-8. The other factors such as BMP-4, Wnt3a, Endothelin 3, and SCF are used in other protocols described in the literature. FGF-8—fibroblast growth factor 8; BMP-4—bone morphogenetic protein 4; Wnt3a—Wnt family member 3A; SCF—stem cell factor. The graphic was prepared using modified elements from Servier Medical Art, found at https://smart.servier.com (accessed on 15 September 2021). (**B**) Schematic presentation of the differentiation protocol, indicating the steps, duration, and markers identified at particular stages. EBs—embryoid bodies; NPC—neuroectodermal progenitors; N1, N2, N3—next stages of differentiation based on specific media N1, N2, N3, respectively; bFGF—basic fibroblast growth factor. For abbreviations of markers identified at each stage, see Table 1 and legend to Figure 3.

The described protocol is a multistep procedure in which piPS cells (protein induced pluripotent stem cells) are sequentially differentiated through embryoid bodies (EB) and progenitor cells to differentiated pigmented cells (Figure 1).

**Figure 2:**
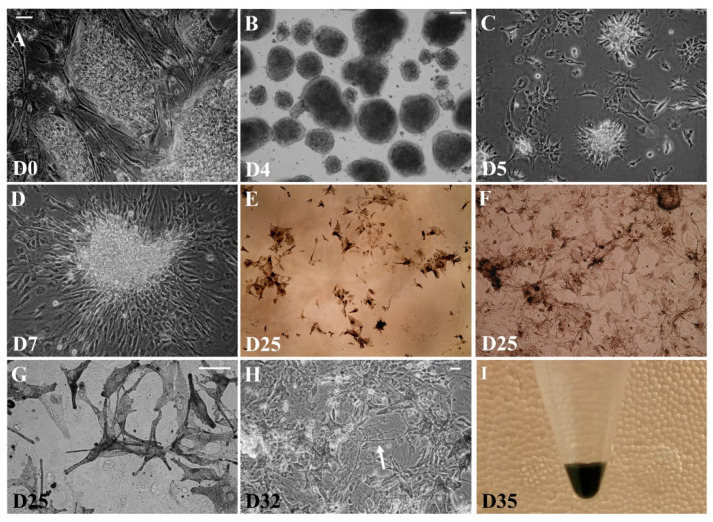
Significant morphological changes during differentiation of pluripotent stem cells (piPS). During the sequence of steps of the previously described differentiation protocol, cells express different morphologies. The piPS cells cultured on a feeder layer (**A**) have typical morphology of embryonic stem cells, that is, small cells with large nuclei, forming compact colonies with distinct borders. Upon culture in suspension, piPS cells form multicellular EBs (**B**). Neuroectoderm progenitor cells are then selected (**C**) and expanded (**D**) in a serum-free medium. Addition of bFGF and FGF-8 stimulates proliferation of progenitors (**E**). Terminal differentiation is achieved on dishes coated with polyornithine and laminin. The final step is sensitive to cell density (compare 0.5 × 10^5^ cells/cm^2^ in panel (**E**) vs. 2 × 10^5^ cells/cm^2^ in panel (**F**)). After 5 days in the terminal differentiating medium, cells become heavily pigmented (**E**–**G**). Black pigment is organized in visible subcellular granules (**G**). The terminal differentiation step delivers a heterogeneous population of cells, among which cells of neuronal morphology can be observed ((**H**, arrow)). Centrifugation of differentiated cells generates a black pellet of cells (**I**). White bar represents 50 µm in (**A**,**C**,**D**); 250 µm in (**B**); and 50 µm in (**G**,**H**).

The morphology of cells changes through each differentiation step, as depicted in Figure 2. In the first step, undifferentiated piPS cells (Figure 2A) are cultured on a feeder layer until high confluency is reached, when they are harvested and seeded into a suspension culture for 4 days, during which multicellular EBs are formed (Figure 2B). This stage is common for many differentiation protocols and comprises the induction of piPS cell differentiation through the suppression of a self-renewal and stemness-supporting environment (more detailed characterization of EBs can be found in the publication of Chlebanowska et al., 2020 [12]). In the next step, neuro-ectodermal progenitors are selected in a serum-free medium (N1 medium) (Figure 2C). The progenitors acquired in this step can be expanded (Figure 2D) and cryopreserved for future differentiation. In the final differentiation step (stage N3), the seeding density is crucial and corresponds to the number of acquired pigmented cells (Figure 2E vs. Figure 2F). Unexpectedly, among the acquired cells, a subpopulation became markedly loaded with black pigment, which appeared to be localized in intracellular granules (Figure 2G). The terminal differentiation step delivers a heterogenic population of cells (Figure 2H). First, pigmented cells appeared in the co-culture as early as on day 5 of terminal differentiation. As we applied a modified dopaminergic neuron differentiation protocol, cells with neuronal morphology could also be observed within the co-culture, although in a low quantity with respect to pigmented cells (Figure 2H, arrow).

### 2.2. Characterization of Pigmented Cells

**Figure 3:**
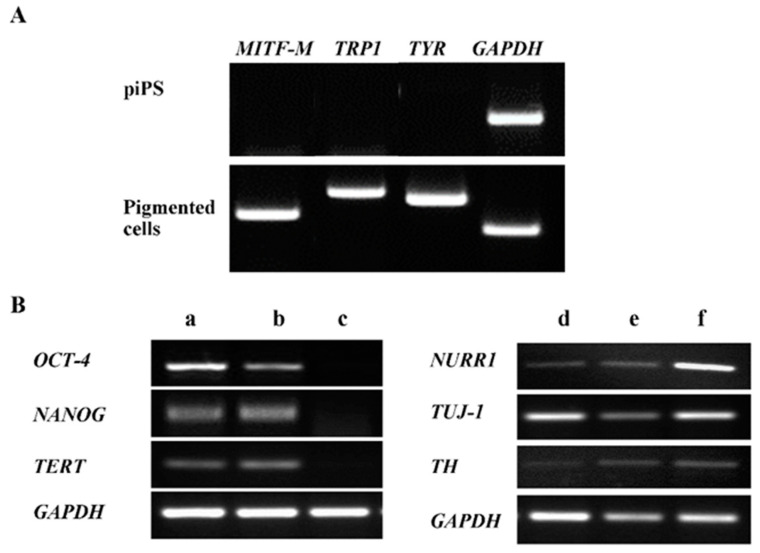
Significant changes in gene expression pattern during differentiation of piPS cells. Expression of several cell types’ markers were analyzed on the level of mRNA by RT-PCR (**A**). Acquired pigmented cells express markers characteristic of melanocytes: MITF-M (microphthalmia-associated transcription factor isoform M), TRP-1 (tyrosinase-related protein 1), and TYR (tyrosinase). (**B**) Letters a–f correspond to appropriate stages of differentiation as visualized in Figure 1: a—piPS, b—EB, c—neuroectoderm progenitor cells after selection, d—progenitor cells after expansion, e—cells after terminal differentiation in low density, f—pigmented cells differentiated in high density. TERT—telomerase; TUJ-1—neuron-specific class III beta-tubulin; TH—tyrosine hydroxylase; NURR1—nuclear receptor-related protein 1; GAPDH—glyceraldehyde 3-phosphate dehydrogenase (housekeeping gene). During neuronal differentiation, the gene expression pattern changed—from embryonic markers (OCT-4—octamer-binding transcription factor 4, NANOG, and TERT) present in piPS cells and EBs to neuron markers (NURR1, TUJ-1, and TH) expressed in terminally differentiated cells.

At each step of differentiation, the expression of selected marker genes was analyzed at the level of their mRNA by RT-PCR (Figure 3). As expected, during the differentiation of piPS cells, some embryonic markers (such as OCT-4, NANOG, and TERT) were downregulated and their expression was not detectable in neuro-ectodermal progenitors (Figure 3B(a–c)). In addition, during the course of cell differentiation, the expression of some neuronal markers (TUJ-1, NURR1, TH) was upregulated (Figure 3B(d–f)), consistent with the presence of some cells having neuronal morphology (Figure 2H). Importantly, in terminally differentiated cells, markers of melanocytes (MITF-M) and melanogenesis (TYR and TRP1) were expressed (Figure 3A). Moreover, there was a low expression of markers characteristic of dopaminergic neurons (TH) in terminally differentiated cells (Figure 3B(f)).

**Figure 4:**
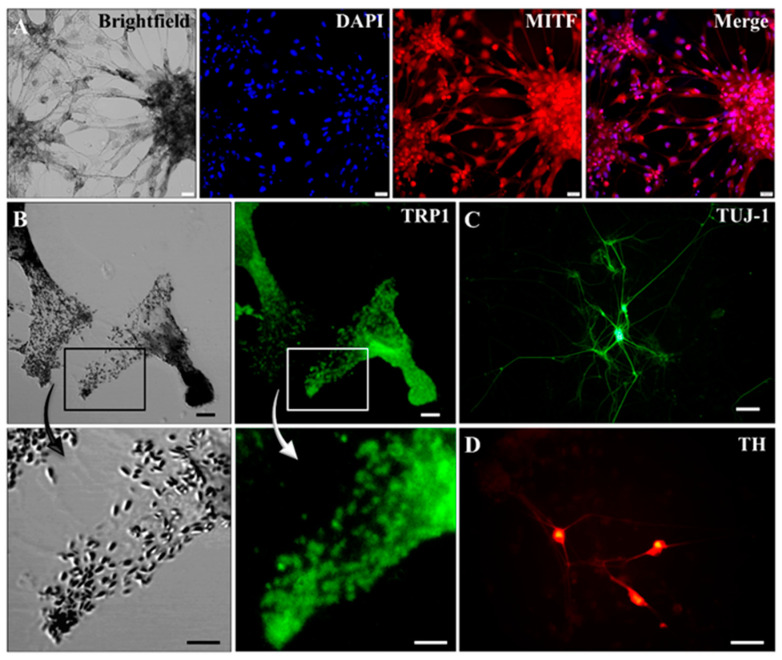
Terminal differentiation in high density produces a heterogeneous population of cells. Differentiation of piPS using the described protocol results in the generation of a heterogeneous cell population: MITF—(**A**) and TRP1-expressing melanocytes (**B**) and TUJ-1—(**C**) and TH-positive dopaminergic neurons (**D**). Notice co-localization of TRP1 and melanin granules in the magnification panels of (**B**). Immunocytochemical staining is shown for TRP-1—tyrosinase related protein 1, MITF—microphthalmia-associated transcription factor, and TUJ-1—neuron-specific class III beta-tubulin, TH—tyrosine hydroxylase. White bar represents 50 µm in (**A**,**B**), 100 µm in (**C**), and 50 µm in (**D**).

Expression of aforementioned markers on the protein level was also analyzed. Differentiated cells expressed TRP1, a marker characteristic of melanocytes that is strongly co-localized with pigment seeds (inset in Figure 4B). Expression of MITF was also detectable, although predominantly in lighter pigmented cells, most likely corresponding to early stages of melanocyte maturation (Figure 4A). Universal neuronal marker TUJ-1 (Figure 4C) was also detected in the co-culture, suggesting the presence of a small proportion of neurons existing in the heterogeneous population of differentiated cells, as previously described (Figure 2H). Among these neurons, a portion proved to be dopaminergic, since they expressed TH (Figure 4D) and produced dopamine, identified by high-pressure liquid chromatography (HPLC, data not shown). These data suggest successful differentiation of piPS cells into pigmented melanocytes and, to a lesser extent, also into neurons.

**Figure 5:**
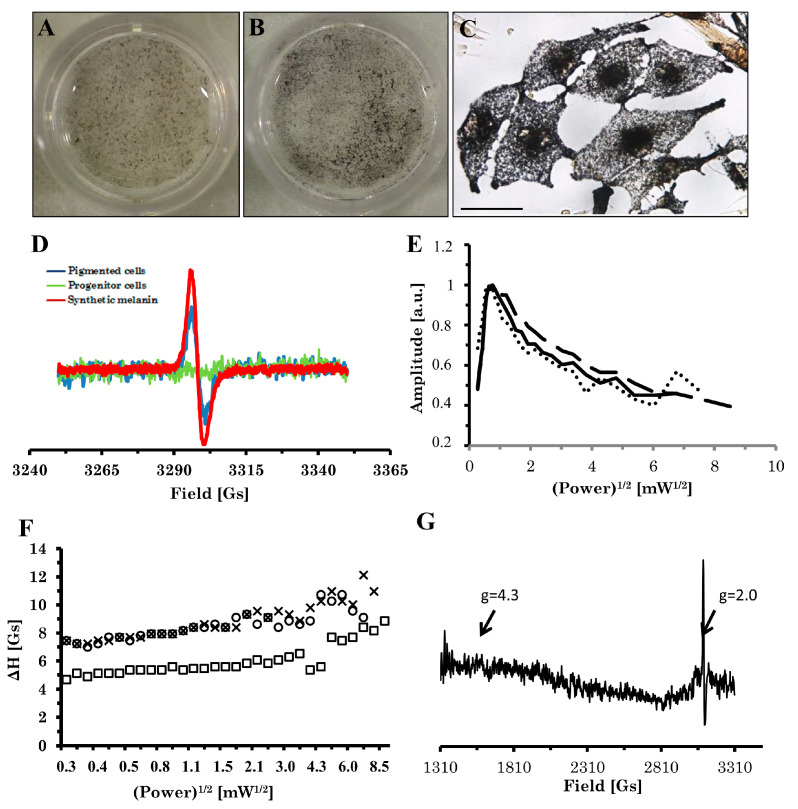
Differentiated cells produce melanin as demonstrated by EPR spectroscopy and staining protocol for melanin pigment. Photographs of differentiated cells: unstained (**A**) and Fontana–Masson-stained (**B**). (**C**) The argentaffinic Fontana–Masson staining revealed an intracellular black net of colloidal silver due to the presence of melanin accumulated in differentiated cells. (**D**) Eumelanin (blue line) was identified by specific EPR signal with no hyperfine splitting, compared with progenitor cells (green line), with lack of signal due to the absence of melanin pigment, and DOPA-melanin (red line) as the reference standard. (**E**–**G**) The chemical and physical composition and structure of the pigment resembled typical melanized tissues, as shown by power saturation of the melanin signal: amplitude (**E**) and linewidth (**F**) of melanin EPR signal versus square root of power for pigmented cells (solid line, circles), murine B16 F10 tissue (dashed line, squares), and pigmented eye bulbs (dotted line, crosses). An EPR spectrum of differentiated pigmented cells registered at a wider field sweep (2000 Gs) revealed no signal of Fe (III) high-spin iron at g = 4.3, which is typical of human neuromelanin ((**G**), [16]), while a strong signal of melanin-free radical is present at g = 2.00. EPR spectrum in (**D**) was measured at room temperature; (**C**) shows a magnification of 400× with a scale bar representing 50 µm. (**E**–**G**) were measured at 77 K.

It is worth stressing that the amount of acquired pigment was incomparably higher than in any observed in vitro culture (Figure 2I shows dark black pellet of acquired cells). Accordingly, we decided to investigate this phenomenon more closely. The intensity of the pigmentation strongly depended on initial cell seeding density prior to the terminal differentiation step. The highest proportion of cells became pigmented when cells were seeded in a density of 2 × 10^5^/cm^2^, suggesting that a larger number of cells in the co-culture corresponds to a higher production of pigment (Figure 5A,B). The black pigment could be observed as early as on the fifth day of terminal differentiation (Figure 2E–G). The most abundant dark pigment in mammals is melanin, present not only in skin and retinal pigment epithelium (RPE) cells but also in the central nervous system as neuromelanin. Skin melanin and brain neuromelanin have different physicochemical properties, as recently reviewed by various publications [13,14,15]. Significant biological differences also occur between melanocytes and RPE cells, which can be observed, e.g., from MITF expression [15].

### 2.3. Electron Paramagnetic Resonance (EPR) Analysis of Pigment

Because melanin is a paramagnetic substance, EPR analysis was performed to identify the pigment (Figure 5D). EPR spectrum of differentiated cell pellets corresponded well to the spectrum of synthetic 3,4-dihydroxyphenylalanine (DOPA)-melanin—a singlet, slightly asymmetric line with g factor ca. 2.004 and linewidth 4.5 Gs, while non-pigmented progenitor cells gave no EPR signal. Specific Fontana–Masson staining additionally confirmed the identity of black pigment as melanin in differentiated cells (Figure 5B,C). The lack of hyperfine structure of the signal supports the view that the type of the observed pigment is closer to eumelanin rather than pheomelanin. Also, the character of the power saturability of the signal (Figure 5E,F) indicates that the observed pigment structurally resembles eumelanin—the signal saturates rapidly at low power and resembles the picture obtained for B16 murine melanoma and eye melanin (Figure 5E). When the EPR spectrum was recorded at a wider field scan, besides a strong melanin signal at g = 2.0, no other signal was detected; in particular, no signal at g = 4.3 was observed, and then the presence of high-spin Fe (III) bound to melanin (Figure 5G) was excluded.

### 2.4. Transmission Electron Microscopy (TEM) and Atomic Force Microscopy (AFM) Characterization of Melanin-Containing Structures

**Figure 6:**
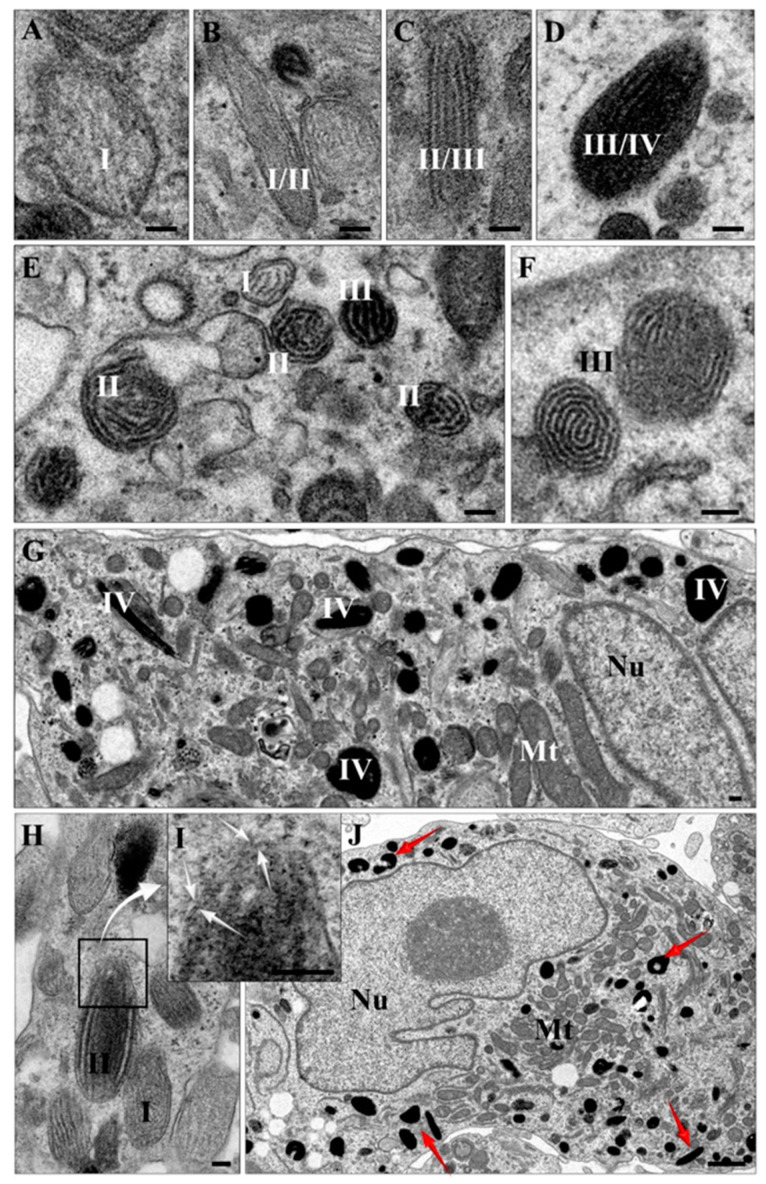
Ultrastructure of melanosomes of pigmented differentiated cells. Melanin in pigmented cells is organized in organelles resembling melanosomes. TEM analysis of differentiated cells reveals sequential stages typical of eumelanosomal maturation, marked as I–IV (**A**–**J**). With the melanosome development, the proteinaceous fibrils become more complex and form a regular network inside the vesicles (**E**,**F**). Their formation begins in Stage I and is completed by Stage II. The first two stages lack pigment and only contain intralumenal proteinaceous fibrils (**A**,**B**,**E**,**F**,**H**). At Stage III and IV, deposition of melanin takes place and results in masking their internal structure. The melanosomes of the final stage (IV) are strongly packed with melanin, visible as an electron-dense material. Strongly pigmented cells contain numerous Stage IV melanosomes evenly distributed in the cytoplasm. The organelles are surrounded by lipid bilayers (**H**,**I**) (indicated by white arrows in high magnification inset (**I**)). The cells contain lobed nuclei and numerous elongated mitochondria (**G**,**J**). Mt—mitochondria; Nu—nucleus; red arrows—melanocytes at Stage IV. Scale bar corresponds to 100 nm in (**A**–**I**) and 1 µm in (**J**).

Physiologically, melanin is organized in specialized organelles called melanosomes in peripheral skin cells [13] or exists as neuromelanin in aged autolysosomal organelles present in neurons with particular features [17,18,19]. To obtain insights into the subcellular localization of melanin in differentiated cells, we performed TEM analysis (Figure 6), which proved that melanin within acquired cells was localized in granules surrounded by lipid bilayers (indicated with arrows in Figure 6I). Moreover, all typical stages of eumelanosome development [20] (named I–IV as shown in Figure 6) could be identified within the co-culture, thus proving active melanogenesis in acquired melanocytes. In particular, they clearly differ from pheomelanosomes, being oval shaped with lamellar ultrastructure and melanin present starting from Stage III [6]. Altogether, these data prove that the acquired cells were able to produce large amounts of melanin and contain it within subcellular organelles resembling melanosomes. Taking the synthetic DOPA-melanin suspension as a standard, we calculated that each measured cell must have contained ca. 31 ± 1.44 pg of melanin. Interestingly, other iPS cell lines subjected to the described protocol (Figure 1) also differentiated into pigmented cells, although to a variable degree (data not shown), proving the well-known fact of strong differences between iPS cell lines, even between cell lines of the same individual [12].

**Figure 7:**
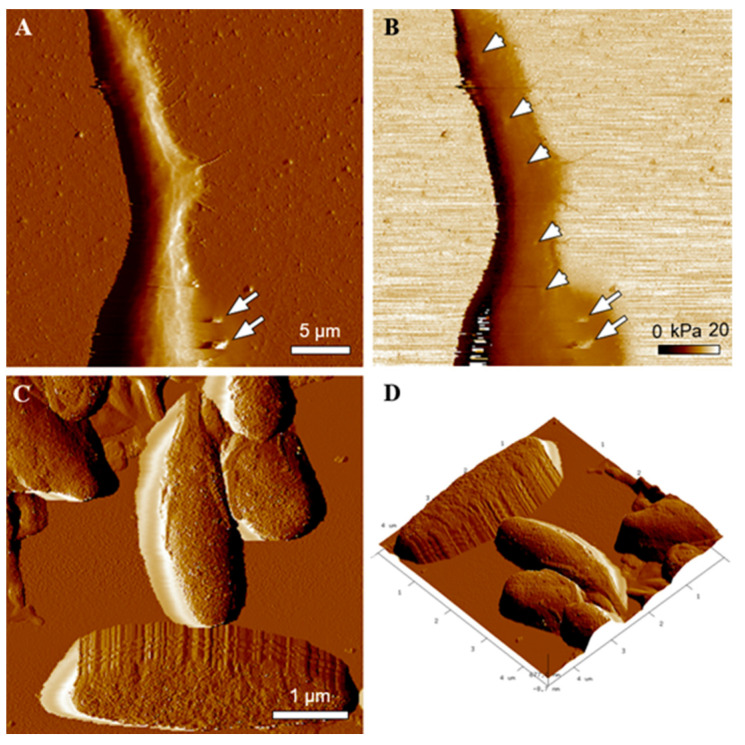
AFM analysis of the differentiated melanocytes and isolated melanosomes. PeakForce error image showing morphology of a melanocytic dendrite (**A**). Arrows indicate melanosomes excreted by the cell. Corresponding elasticity map of Young’s modulus values of the same area (**B**). Arrow heads indicate melanin granules in the differentiated melanocyte. Note that high values of Young’s modulus in the melanocyte correspond to the positions of the pigment granule in the cell. PeakForce error image showing high resolution morphology of isolated melanosomes (**C**) and of 3D topography of the pigment granules (**D**).

Most recently, melanosomes were recognized as “hard like rock” organelles that dramatically modify rheological properties of pigmented cells, including melanocytes [21,22]. This unexpected property of melanosomes has a significant effect on the function of pigmented cells (e.g., [23,24,25]). Thus, we examined rheological properties of both piPS-derived melanocytes and isolated melanin granules employing AFM. Figure 7 shows a high resolution AFM image of a melanocytic dendrite (Figure 7A) with melanosomes excreted by the cell (indicated by arrows). Moreover, an elasticity map of the cell (Figure 7B) shows co-localization of high values of Young’s modulus with the positions of the pigment granules in the cell. Figure 7 also shows high resolution AFM images of the morphology and 3D topography of isolated melanosomes from the cells (Figure 7C,D). As evident, the morphology of the granules is typical of eumelanosomes, as indicated in previous work [26].

## 3. Discussion

Differentiation of iPS cells is a multistep process, in which the pattern of gene expression is converted from that which is typical of embryonic (pluripotent) cells to one that is specific for adult cells [27]. In our study, we observed such a transition by means of mRNA levels. Downregulation of OCT-4, NANOG, and TERT suggest a decrease in stem cell potential during differentiation. The appearance of TYR, TRP1, and MITF-M as well as TUJ-1, NURR1, and TH expression suggests the presence of two different cell types (heterogenous co-culture) among differentiated cells: subpopulations of the pigmented cells (vast majority) and dopamine-secreting neurons (minority). Admixture of dopaminergic cells generated during differentiation has been described previously [17]; however, it did not involve the appearance of pigmented cells.

The most important result is the appearance of cells filled with dark pigment during the course of terminal differentiation. This observation was highly repetitive (n = 10). The black pigment was unambiguously identified as melanin by EPR and Fontana–Masson staining in differentiated cells. Quantitative analysis using EPR allowed us to estimate the melanization of the cells. As the pooled pellet revealed high heterogeneity, the actual content of the pigment in a fully differentiated pigmented cell must have been even higher than 31 ± 1.44 pg/cell. For comparison, popular cell lines of normal murine melanocytes melan-a and melan-b contain 65 ± 4.7 and 71 ± 1.85 pg/cell, respectively (detected by EPR) [28]. By contrast, murine melanoma B16 reveals very variable melanization, e.g., 1.06 ± 0.59 pg/cell (data obtained from wet tissue using chemodegradation followed by the PTCA/AHP HPLC method) [29], 6.17 ± 4.25–34.66 ± 11.295 pg/cell [30] (EPR, control silencing of PPARα), and up to 133.3 ± 38.2 pg/cell [31] (EPR, 72h incubation in DMEM). This places our differentiated piPS cells among relatively highly pigmented ones.

The character of the power saturation curves of the EPR signal and lack of high-spin iron(III) EPR signal at g = 4.3 strongly suggest that the structure and composition of our melanin is similar to that of more typical pigmented cells (the “classical” pigmented B16 melanoma and the abovementioned RPE cells); i.e., there was no change in the character of the saturation curve, which might have suggested interactions with metal or a stronger degree of pigment degradation [16] or transformation, which would be characteristic of neuromelanin [32,33,34,35]. Electron microscopy studies revealed a characteristic subcellular organization of melanin within membranous organelles, with a morphology similar to eumelanosomes at different stages [6,20]. In this study, we have observed the four steps of melanosome formation and maturation. In Stage I, the intralumenal proteinaceous fibril formation takes place, which are completely shaped by the end of Stage II. The melanosomes of Stages I and II are ellipsoidal and do not contain pigment; thus, the ultrastructure of the fibrils is clearly visible in electron microscopy [6] (this study). Melanin synthesis and its deposition in Stage III results in a strong pigmentation at Stage IV, when melanosomes are completely filled with melanin and the organization of fibrils is masked [6,20].

In addition, AFM analysis confirmed that the morphology of the pigment granules was typical of eumelanosomes [26]. The obtained results indicated that the pigment granules modify nanomechanical properties of differentiated melanocytes. This may have a significant impact on the biomechanical function of the differentiated melanocytes, which should be addressed in detail in future work. Thus, pigmented cells acquired in this study resemble melanocytes with respect to numerous features. They possess their characteristic marker expression (TRP1, MITF-M, TYR), both on the level of mRNA and protein. The presence of markers such as TYR and TRP1 suggests active melanogenesis in acquired cells, and the existence of all characteristic stages of melanosomes maturation further proves that the generated cells are functional melanocytes [6,7], although their morphology might not be entirely characteristic of melanocytes. This may be due to the presence of other cell types within the heterogeneous cell population or the differentiation process and culture conditions, which are more characteristic for neurons than melanocytes. Intercellular communication and culture conditions are known to strongly affect cell morphology as well as the morphology of intracellular melanosomes.

The appearance of melanocytes upon neuronal protocol of differentiation of iPS cells might seem surprising at first sight. However, it becomes more logical when one looks at the origin of melanocytes and neurons. Both of these cell types originate from ectoderm during ontogenesis [36]. Thus, it is not entirely unexpected to acquire melanocytes during what was supposed to be a neuronal differentiation protocol. This means that in the stage of pattern formation, the cells have a multipotent common stage, i.e., delivering both melanocytes and neurons. This phenomenon was probably achieved by our modifications in the described protocol [11]. Differentiation of iPS cells in specific conditions (high cell density, medium composition) may have resulted in the generation of dopaminergic neurons with an efficacy much lower than expected; however, we obtained a highly enriched subpopulation of cells able to very efficiently produce melanin.

One may consider that described protocols for iPS differentiation towards melanocytes involve treatment with differentiating media of compositions other than that applied here (e.g., cholera toxin, MSH, etc.) [8,9,10]. We speculate that FGF-8, added to the N2 medium, is a key factor in determining the efficiency of our protocol. Comparing with other published protocols [8,9,10,37,38,39], we have found that melanocyte induction media contain, e.g., BMP-4, Wnt3a, Endothelin, or SCF, but none of them were supplemented with FGF-8. FGF-8 plays an important role in the regulation of embryonic development, cell proliferation, and cell migration and differentiation, especially the differentiation of oligodendrocyte progenitor cells [40]. It has been shown that during embryogenesis, FGF-8 expression increases in neuroepithelium, which is a common precursor for midbrain and pigmented cells (i.e., RPE cells) [41]. This might indicate the role of FGF-8 as the main factor that is responsible for differentiation towards highly pigmented cells.

We speculate that the level of the final differentiation might be an effect of differences between iPS cell lines and their ability to efficiently differentiate toward specific cell types upon different treatment. Applying a iPS cell line appeared exceptionally responsive to the differentiation protocol used, delivering a high number of melanocytes, while others were not so responsive, generating only modest pigmentation (data not shown). Strong differences between iPS cell lines in their epigenetic landscape and differentiation efficacy are well described [12,27,42,43,44]. We considered other possible explanations for the presence of dark pigment in our co-cultures despite melanocyte generation. Other cell types containing high levels of dark pigment are retinal pigmented epithelium (RPE) cells and neuromelanin-loaded neurons in substantia nigra and locus coeruleus. The first cell type has even been reported as a common by product of dopaminergic differentiation of iPS cells [45,46]. However, generation of this cell type is highly unlikely, as epithelial morphology of RPE cells is significantly different from acquired pigmented cells, and immunocytochemical staining for RPE markers gave negative results (data not shown). The neuromelanin formation can be excluded, as the morphology of a neuromelanin-containing organelle is completely different from melanosome [19,20] and also includes lipid bodies. Moreover, the synthesis of neuromelanin likely does not require TYR [19,47,48], while in our cells, this enzyme is highly expressed.

To our knowledge, this is the first report showing that an in vitro cell culture can generate such quantities of melanin with features of natural melanins. Previous data showed only modest production of melanin in cell cultures, in which melanocytes were identified by their more or less specific antigens rather than strong pigmentation [7,20,37]. Moreover, our model may be helpful for establishing to what degree neuromelanogenesis and eumelanogenesis have common aspects. We believe that the described protocol may generate a new common source of melanocytes and melanin in vitro, which may ease research on biophysical properties of melanin and biological functions of melanocytes in pigmentation disorders.

## 4. Materials and Methods

### 4.1. Cell Culture and EBs Formation

The piPS (System Biosciences, Palo Alto, CA, USA [49]) were cultured on a feeder layer of mouse embryonic fibroblasts (MEFs) in a medium consisting of DMEM/F12 with 20% KSR (both from ThermoFisher Scientific, Waltham, MA, USA), 2 mM GLUTAMAX, 100 µM β-mercaptoethanol (Sigma-Aldrich, St. Louis, MO, USA), 100 µM Non-Essential Amino Acids, 100 U/mL/100 µg/mL Penicillin/Streptomycin, and 10 ng/mL bFGF (all from ThermoFisher Scientific, Waltham, MA, USA), until confluent. To induce EB formation, piPS cells were rinsed with PBS without Ca^2+^ and Mg^2+^ (Lonza, Salisbury, MD, USA), dissociated by Accutase (ThermoFisher Scientific, Waltham, MA, USA), and plated at a density of 2.5–5 × 10^4^ cells/cm^2^ in the medium described above supplemented with Y27632 and without bFGF. The EBs were formed for 4 days and then plated onto an adhesive dish (of the same size as the non-adhesive dish used) in a DMEM medium (Lonza, Salisbury, MD, USA) containing 10% FBS, 4.85 g/L glucose, 2 mM L-glutamine, and 100 U/mL/100 µg/mL Penicillin/Streptomicin.

#### Differentiation in N1, N2, and N3 Media

During the differentiation, three types of media were used: N1, N2, and N3. Each of them contains N-2 supplement (ThermoFisher Scientific, Waltham, MA, USA), which is composed of human transferrin 10 000 µg/mL, recombinant human insulin 500 µg/mL, progesterone 0.63 µg/mL, putrescine 1611 µg/mL, and sodium selenite 0.52 µg/mL. The media were changed every 2 days.

After 24 h, the selection of progenitor cells was initiated by replacing the culture medium with N1 medium, containing DMEM/F12 medium, 1xN-2 Supplement, 250 ng/mL Fibronectin, and 100 U/mL/100 µg/mL Penicilin/Streptomicin (all from ThermoFisher Scientific, Waltham, MA, USA) for 6–10 days. Next, cell expansion was performed. Selected cells were dissociated by 0.05% trypsin/0.04% EDTA (Lonza, Salisbury, MD, USA) and plated on dishes pre-coated with polyornithine and laminin (both from ThermoFisher Scientific, Waltham, MA, USA) at a density of 2 × 10^5^ cells/cm^2^ in N2 medium, containing DMEM/F12, 1xN-2 Supplement, 20 µg/mL bFGF, 100 ng/mL FGF-8 (all from ThermoFisher Scientific, Waltham, MA, USA), and 1 mg/mL laminin (R&D Systems, Mineapolis, MN, USA). Selected cells were expanded for 6–10 days until 80–90% confluency was reached. Terminal differentiation was induced by the removal of FGFs. The medium was replaced with differentiation medium N3, which consisted of 1xN-2 Supplement, 1 mg/mL laminin, 0.5 mM dibutyryl-cAMP, 200 µM Ascorbic Acid (both from Sigma-Aldrich, St. Louis, MO, USA), and 100 U/mL/100 µg/mL Penicillin/Streptomycin. The cells were differentiated for 6–15 days until they became heavily pigmented.

### 4.2. RT-PCR

Gene expression on the level of mRNA was analyzed by RT-PCR. Briefly, total RNA was isolated with GeneMATRIX Universal RNA kit (Eurx, Gdańsk, Poland). Reverse transcription (RT) was performed with MMLV reverse transcription kit (Promega, Madison, WI, USA) according to manufacturer instructions. PCR reactions were performed with Taq Master Mix kit (Eurx, Gdańsk, Poland); 100 ng of generated cDNA was used per reaction. All primers used were designed to work optimally at an annealing temperature of 55 °C. Primer sequences have been collected in the Table 1.

### 4.3. Immunocytochemistry

Cells were rinsed with PBS with Ca^2+^ and Mg^2+^ and fixed in 4% paraformaldehyde (20 min, room temperature). Next, cells were rinsed with PBS with Ca^2+^ and Mg^2+^ and permeabilized with 0.1% Triton X-100 in PBS with Ca^2+^ and Mg^2+^ (5 min, room temperature). Then, unspecific binding sites were blocked with 3% BSA in PBS with Ca^2+^ and Mg^2+^ (30 min, room temperature). The cells were incubated with appropriate antibodies diluted in 3% BSA in PBS with Ca^2+^ and Mg^2+^ overnight at 4 °C, rinsed with PBS with Ca^2+^ and Mg^2+^, and incubated with fluorescently labeled secondary antibodies and Hoechst 33,342 (Life Technologies, Carlsbad, CA, USA) diluted in 3% BSA in PBS with Ca^2+^ and Mg^2+^ for 1 h at room temperature in the dark. Antibodies and dilutions were as follows: MITF, mouse 1:1000; TRP1, mouse 1:1000; TH, rabbit 1:1000; TUJ-1, mouse 1:100 (all from Millipore, Burlington, MA, USA), goat anti-rabbit-Alexa555, 1:200, goat anti-mouse-Alexa488 1:200 (both from ThermoFisher Scientific, Waltham, MA, USA). Specimens were photographed with an inverted fluorescent microscope (Olympus BX90, Shinjuku, Tokio, Japonia) and with a scanning laser confocal microscope from Zeiss (LSM900 with AiryScan 2.0) (Jena, Germany). Images were generated using ZEN 3.1 software (Zeiss).

### 4.4. Modified Fontana–Masson Staining for Melanin

We modified a previously published procedure [50] to stain intracellular melanin directly in the culture dishes (plastic multi-well plates). Briefly, the plates with adhered, differentiated cells were washed in warm (37 °C) PBS with Ca^2+^ and Mg^2+^ ions and fixed on ice with cold (−20 °C) methanol (POCh, Gliwice, Poland) for 5 min, rinsed in distilled water, and incubated in previously warmed to 56 °C silver solution for 30 min at 56 °C in the dark. The control cells were incubated in PBS at room temperature. The silver solution was prepared as described previously [50]. After staining and washing in distilled water, the cells were incubated in 5% sodium thiosulphate (POCh, Gliwice, Poland) (1 min), flushed with tap water (3 min), counterstained (0.5% neutral red, 10 min), flushed with tap water again, dehydrated in the ethanol series (70.96 and 100% *v*/*v*, 2× each), and dried. The images were recorded with an Eclipse TS 100 microscope equipped with a Nikon digital camera D7000 and Control Pro2 imaging software (Nikon Corporation, Tokyo, Japan). Macrophotographies were recorded with a Nikon digital camera D7000 equipped with Nikkor Micro AF 60 mm 1:2.8 lenses (Nikon).

### 4.5. EPR Measurements

The cells were measured as pellets at room temperature and at 77K. The parameters of measurement at room temperature were as follows: field 3300 ± 50Gs, modulation 100 kHz, 5Gs, and power 2.9 mW. The pellet of cells was measured in a Pasteur pipette and contained ca. 2,000,000 cells (volume of 20 μL). We also measured an equivalent sample of DOPA-melanin under the same conditions (a generous gift synthesized by Prof. Andrzej Żądło using tyrosinase according to d’Ischia et al. [37], containing 17.8 ± 0.14 mg/mL). The spectra were gained in a digital form (acquisition time of 200 s, time constant of 0.1 or 0.3 s registered 2 times and averaged) and compared.

The amount of melanin was estimated by taking a sample of DOPA melanin as a standard [28].

The uncertainty of the melanin measurement was estimated using the method of estimation of square error propagation (see: JCGM100: 2008, SèvresFrance; http://www.bipm.org/utils/common/documents/jcgm/JCGM_100_2008_E.pdf) (accessed on 19 June 2021).

For measurements in liquid nitrogen, we used standard icicles of pellets (diameter of 4 mm) of 200 μL in volume and equivalent samples containing murine eyes (in toto) and solid tissues of murine B16-F10 pigmented melanoma. The eyes were obtained post mortem as organs, without additional treatment, and the B16 tumor was a byproduct of an experiment performed for some other reasons (the Local Ethical Committee for Animal Experimentation, Kraków, Poland; decision No. 76/2011, 8 June 2011). The saturation curves were prepared under similar conditions as measurements at room temperatures, except for modulation amplitude (10 Gs) and temperature (77 K). The amplitudes were normalized to 1 for the maximal amplitude of each sample. We also registered the changes in the linewidth according to the changing power. The measurements aimed at Fe (III) (g-4.3) were performed at field 2310 ± 1000 Gs, modulation 10 Gs, power 2.9 mW, and gain 500,000 (3 × 300 s, at time constant 0.1s and averaged thereafter). All the measurements were performed using a Varian E3 X-band EPR spectrometer (Sunnyvale, LA, USA) with a rectangular T102 resonant cavity.

The melanin assay was repeated three times in total, and each time, the full number of cells obtained over one procedure of cultivation was pooled.

### 4.6. TEM Imaging

Terminally differentiated cells were fixed in a monolayer with 2.5% solution of glutaraldehyde in PBS with Ca^2+^ and Mg^2+^ (pH 7.3) for 3 h at 4 °C and then scraped with a cell scraper. After centrifugation, fixed cell pellets were covered with fresh glutaraldehyde solution and stained in 1% solution of osmium tetroxide. In the next steps, cells were rinsed and dehydrated in ethanol series (50%, 70%, 90%, 95%, 100%) and embedded in epoxy resin Epon 812 (Serva, Germany). The ultrathin sections (90 nm thick) were contrasted with 2% uranyl acetate and lead citrate and analyzed and photographed under a Jeol JEM 2100 transmission electron microscope at 80 kV.

### 4.7. AFM Imaging and Elasticity Measurements

AFM analysis was conducted using a Bioscope Catalyst AFM (Bruker, Karlsruhe, Germany) coupled with an inverted optical microscope AxioObserver Z1 (Zeiss, Jena, Germany). Melanocytes were examined in culture medium at 37 °C, whereas melanosomes were investigated in PBS buffer at room temperature. Both cells and pigment granules were analyzed employing the PeakForce Tapping mode using the ScanAsyst-Fluid probe with a nominal tip radius of 20 nm and an experimentally determined spring constant of 0.68 N/m (Bruker Probes, Karlsruhe, Germany). During the analysis of cells, the PeakForce Capture was turned on, which resulted in the acquisition of force curves from each pixel of an image. To calculate the values of Young’s modulus in the elasticity map, the obtained force curves were analyzed using AtomicJ software [51]. In brief, the recorded force–displacement curves were converted into force–indentation curves and fitted with the Sneddon model. A detailed description of the analysis can be found elsewhere [52].

## 5. Conclusions

Our new protocol is extremely efficient in generating melanin-producing cells. It also differs significantly from other protocols described in the literature. As melanin itself always comes in short supply and with low purity for downwards studies, our results may pave the way for the generation of unlimited, on-demand, in vitro sources of human melanin. Pigmented cells may serve as an in vitro model for studying pathomechanisms of pigmentation disorders such as vitiligo. Specific differentiation of iPS cells obtained from different patients will allow us to use this method to develop personalized therapies.

## Figures and Tables

**Table 1:** Primer sequences (from 5′ to 3′) used for RT-PCR gene expression analysis. MITF—melanocyte-specific microphthalmia-associated transcription factor isoform M; TRP1—tyrosinase related protein 1; TYR—tyrosinase; OCT-4—octamer-binding transcription factor 4; TERT—telomerase reverse transcriptase; GAPDH—glyceraldehyde-3-phosphate dehydrogenase; TUJ-1—neuron-specific class III beta-tubulin; TH—tyrosine hydroxylase; NURR1—nuclear receptor-related 1 protein.

Gene	Forward Primer	Reverse Primer
MITF-M	ACCTTCTCTTTGCCAGTCCATCT	GGACATGCAAGCTCAGGACT
TRP1	TGTAACAGCACCGAGGATGG	TGTCCAATAGGGGCATTTTCCA
TYR	GAATGCTCCTGGCTGTTTTG	AGTCGTCTCTCTGTGCAGT
OCT-4	ATGGCGGGACACCTGGCTT	GGGAGAGCCCAGAGTGGTGACG
Nanog	TGAACCTCAGCTACAAACAG	TGGTGGTAGGAAGAGTAAAG
TERT	TGTGCACCAACATCTACAAG	GCGTTCTTGGCTTTCAGGAT
GAPDH	CAAAGTTGTCATGGATGACC	CCATGGAGAAGGCTGGGG
TUJ-1	TGGATTCGGTCCTGGATGTG	ACCTTGCTGATGAGCAACG
TH	GAGTACACCGCCGAGGAGATTG	GCGGATATACTGGGTGCACTGG
NURR1	CCCAGTGGAGGGTAAACTCA	AATGCAGGAGAAGGCAGAAA

## Data Availability

Data is contained within the article.
